# Altered brain functional activity and connectivity in bone metastasis pain of lung cancer patients: A preliminary resting-state fMRI study

**DOI:** 10.3389/fneur.2022.936012

**Published:** 2022-09-21

**Authors:** Daihong Liu, Xiaoyu Zhou, Yong Tan, Hong Yu, Ying Cao, Ling Tian, Liejun Yang, Sixiong Wang, Shihong Liu, Jiao Chen, Jiang Liu, Chengfang Wang, Huiqing Yu, Jiuquan Zhang

**Affiliations:** ^1^Department of Radiology, Chongqing University Cancer Hospital, School of Medicine, Chongqing University, Chongqing, China; ^2^Department of Palliative Care and Department of Geriatric Oncology, Chongqing University Cancer Hospital, School of Medicine, Chongqing University, Chongqing, China

**Keywords:** bone metastasis pain, resting-state fMRI, low-frequency fluctuations, regional homogeneity, functional connectivity

## Abstract

Bone metastasis pain (BMP) is one of the most prevalent symptoms among cancer survivors. The present study aims to explore the brain functional activity and connectivity patterns in BMP of lung cancer patients preliminarily. Thirty BMP patients and 33 healthy controls (HCs) matched for age and sex were recruited from inpatients and communities, respectively. All participants underwent fMRI data acquisition and pain assessment. Low-frequency fluctuations (ALFF) and regional homogeneity (ReHo) were applied to evaluate brain functional activity. Then, functional connectivity (FC) was calculated for the ALFF- and ReHo-identified seed brain regions. A two-sample *t-*test or Manny–Whitney *U*-test was applied to compare demographic and neuropsychological data as well as the neuroimaging indices according to the data distribution. A correlation analysis was conducted to explore the potential relationships between neuroimaging indices and pain intensity. Receiver operating characteristic curve analysis was applied to assess the classification performance of neuroimaging indices in discriminating individual subjects between the BMP patients and HCs. No significant intergroup differences in demographic and neuropsychological data were noted. BMP patients showed reduced ALFF and ReHo largely in the prefrontal cortex and increased ReHo in the bilateral thalamus and left fusiform gyrus. The lower FC was found within the prefrontal cortex. No significant correlation between the neuroimaging indices and pain intensity was observed. The neuroimaging indices showed satisfactory classification performance between the BMP patients and HCs, and the combined ALFF and ReHo showed a better accuracy rate (93.7%) than individual indices. In conclusion, altered brain functional activity and connectivity in the prefrontal cortex, fusiform gyrus, and thalamus may be associated with the neuropathology of BMP and may represent a potential biomarker for classifying BMP patients and healthy controls.

## Introduction

Pain is the most frequent symptom of cancer survivors, and this symptom has increased in prevalence (1). A previous review revealed that 64% of cancer patients reported pain (2). Bone metastasis pain (BMP) is one of the most prevalent sources of pain since common cancers, including lung, breast, and prostate cancers, have a propensity to metastasize to multiple bones (3). Existing treatments for BMP may be ineffective and fraught with side effects. Therefore, understanding the pathophysiology of BMP may guide the search for novel intervention options.

The brain is necessary for the multidimensional pain experience, and activity in multiple brain regions has been found to be associated with noxious stimuli. Therefore, brain imaging, such as functional magnetic resonance imaging (fMRI), provides valuable information. Among the fMRI indices, the amplitude of low-frequency fluctuations (ALFF) is used to evaluate brain functional activity (4), and regional homogeneity (ReHo) is used to characterize the synchronization of fluctuations of a voxel with its neighboring voxels (5). For instance, ALFF was reported to be increased in the post- and precentral gyrus, paracentral lobule, supplementary motor area, and anterior cingulate cortex, and this feature may be associated with the neuropathology of chronic low back pain (6). ReHo was decreased in the thalamus in neuropathic pain (7), and ReHo values in abnormal brain regions were associated with pain intensity in postherpetic neuralgia patients (8). In addition, functional connectivity (FC), which is used to assess brain activity synchronization between any set of brain areas, was reported to be increased in the insular cortex in our previous study on trigeminal neuralgia (9). The combination of ALFF, ReHo, and FC was also used to explore the brain activity of migraine (10), visceral pain (11), and other types of pain. However, the signature of activation may vary with the different types of nociceptive stimulation (12). The brain functional abnormalities of BMP remain unclear.

The mechanisms of BMP have been investigated in previous studies. Regarding molecular mechanisms, BMP is associated with inflammatory mediators, a highly acidic environment, and cancer invasion of peripheral nerve endings (13). Then nociceptive signals are transmitted to the cerebral cortex and subcortical structures *via* the dorsal horn of the spinal cord. Thus, the sensory, cognitive, and affective aspects of pain experience will be processed within the brain. For instance, the primary somatosensory cortex and thalamus receive nociceptive input from the spinal cord and encode the intensity of pain. In addition, nociceptive signals are sent to the brainstem, midbrain, and medullary areas, where they might modulate the perception and sensation of a noxious stimulus (14, 15). In neuroimaging studies, the cingulate cortex, prefrontal cortex, and ventral striatum showed pain-specific FC changes in a mouse model of metastatic bone cancer (16). Furthermore, prospective administration of anti-nerve growth factor treatment can prevent pain-induced FC adaptations in ascending and descending pain pathways in the same mouse model (17). Animal models of BMP provide invaluable information for pain pathophysiology; however, brain functional activity and connectivity have not been reported in the human population.

Here, we aimed to preliminarily explore the brain functional activity and connectivity patterns in BMP. ALFF and ReHo were applied to evaluate brain functional activity. Then, the FC was calculated for the ALFF- and ReHo-identified seed brain regions. Finally, the discrimination performance of neuroimaging indices was assessed to investigate whether these changes are useful for classifying BMP. The findings may advance our understanding of the mechanism of BMP and potentially provide neuroimaging biomarkers.

## Materials and methods

### Subjects

From August 2020 to November 2021, thirty-six lung cancer patients with BMP were recruited from inpatients, and 36 healthy controls (HCs) matched for age and sex were recruited from communities. Inclusion criteria: (1) lung cancer patients suffering from bone metastasis pain, (2) the BMP patients can tolerate the MRI scan and (3) right-handed. BMP patients were diagnosed with lung cancer according to pathology and bone metastasis according to medical imaging and/or pathology. Subjects with brain structural abnormalities, neurological or psychiatric diseases, disability, left-handedness, and contraindications to MRI examination were excluded prior to enrollment. The experiment was approved by the Medical Research Ethics Committee of Chongqing University Cancer Hospital (Chongqing, China). All participants provided written informed consent before the experiment. All procedures were performed in accordance with the approved study protocol.

According to the head motion profile derived from subsequent MRI data processing, six BMP and three HCs with head motion >2 mm in any direction or 2° at any angle were excluded. Therefore, 30 BMP patients and 33 HCs were involved in the study. Demographic information, including age and sex, was obtained across the two groups. The intensity of real-time pain was obtained according to patients' feelings on a visual analog scale (VAS). All subjects underwent evaluation of anxiety and depression using the Self-Rating Anxiety Scale (SAS) and Self-Rating Depression Scale (SDS). Details are shown in Table 1.

**Table 1:** Demographic and neuropsychological data comparisons.

	**BMP patients**	**Healthy controls**	***p*-Values**
Age (years)	59.57 ± 9.66	59.55 ± 9.38	0.993
Sex (male/female)	21/9	22/11	0.777^a^
Disease duration (days)	240.00 (180, 330)	NA	NA
Pain score	2 (1, 3)	NA	NA
SAS	35.70 ± 9.48	33.97 ± 6.30	0.393^b^
SDS	32.00 (25.50, 39.00)	31.00 (25.50, 39.00)	0.809^c^

Data are presented as n for proportions, means ± SD for normally distributed continuous data, and median (QR) for nonnormally distributed data.

a The *p* value for sex was obtained using the χ^2^ test.

b The *p* value was obtained using the two-sample *t*-test.

c The *p* value was obtained using the Mann–Whitney *U*-test.

BMP, bone metastasis pain; SAS, self-rating anxiety scale; SDS, self-rating depression scale; NA, not applicable.

### MRI scan protocol

Soon after the pain evaluation, structural and functional MRI scanning were performed with a 3.0 T scanner (Magnetom Prisma; Siemens Health Care, Erlangen, Germany) using a 64-channel head-neck coil. Earplugs were used to alleviate the influence of noise and cushions to restrict head motion. Subjects stayed awake with their eyes closed and were instructed to not think about any topics during the scanning.

Conventional T2-weighted images and fluid-attenuated inversion recovery (FLAIR) images were acquired for radiological evaluation of brain structural abnormalities. Then, sagittal T1-weighted structural images were acquired using volumetric 3D magnetization prepared by a rapid-acquisition gradient-echo (MP-RAGE) sequence: repetition time/echo time = 2,100 ms/2.26 ms, flip angle = 8°, field of view = 256 × 256 mm^2^, slices = 192, thickness = 1 mm, matrix = 256 × 256 and voxel size = 1 × 1 × 1 mm^3^, for a total of 4 min and 53 s. Resting-state functional images were acquired transversely using an echo planar imaging (EPI) sequence: repetition time/echo time = 2,000 ms/30 ms, flip angle = 70°, field of view = 240 × 240 mm^2^, slices = 36, thickness = 3 mm, matrix = 80 × 80, voxel size = 3 × 3 × 3 mm^3^, and 240 volumes with a total of 8 min and 8 s.

### MRI data processing

Conventional images obtained with anatomical scans were reviewed by two radiologists with at least 5 years of experience in neuroradiology, and no subjects were excluded for brain abnormalities. The MRI data were processed with a standard protocol in DPABI V6.0 (http://rfmri.org/) (18). (1) Data in DICOM format was converted to NIfTI format. (2) For magnetization equilibrium, the first 10 volumes of individual resting-state functional images were removed. (3) Slice timing was used to correct the remaining 230 volumes due to the temporal offset between slices. (4) Realignment was performed to correct for head motion so that the brain across images was in the same position. According to the realignment parameters, a report of head motion was automatically generated for subject exclusion with head motion of >2 mm in any direction or 2° at any angle. (5) In covariate regression, the Friston 24-parameter model was applied to regress out head motion effects. Other nuisance variables including white matter signal and cerebrospinal fluid signal were regressed out by using Statistical Parametric Mapping's a priori tissue probability maps (empirical thresholds: 90% for white matter mask and 70% for cerebrospinal fluid mask). Global brain signal regression was not performed because it can cause correlation coefficient redistribution and ambiguous interpretation of negative correlations of FC. (6) To make inter-subject comparisons feasible, individual functional images were warped to standard Montreal Neurological Institute (MNI) space through spatial normalization. (7) Detrending was applied to reduce the systematic signal drift with time using a linear model. (8) Finally, to reduce the effects of very-low-frequency and high-frequency physiological noise, the data were band-filtered (0.01–0.10 Hz).

Then, ALFF and ReHo were calculated and standardized with the mean division to reduce the impact of many sources of nuisance variation and increase the test–retest reliability (19). Specifically, ALFF is calculated as the sum of amplitudes within a specific low-frequency range (0.01–0.10 Hz) after transforming voxel time series frequency information into the power domain with a fast Fourier transform (4). ReHo is calculated as Kendall's coefficient of concordance (KCC) among a seed voxel and its neighbor 26 voxels (5). The widely used mean division is to calculate the mean across voxels for neuroimaging indices and divide the value at each voxel by the mean within gray matter. Smoothing (with a 6 mm full-width half-maximum isotropic Gaussian kernel) was conducted before the ALFF calculation and after the ReHo calculation. Brain regions with altered ALFF or ReHo in BMP patients were set as seeds to obtain the averaged time course, and calculate their *Pearson* correlation coefficient values for the remainder of the whole-brain voxels. Fisher's *r*-to-*z* transformation was applied to normalize the distribution correlation coefficient to obtain FC.

### Statistical analysis

Demographic and neuropsychological data were analyzed with SPSS software (version 25.0; IBM Corp., Armonk, NY, USA). Two-sample *t-*tests or Mann–Whitney *U*-tests were applied to compare the data between two groups according to the distribution checked with the Kolmogorov–Smirnov test. The chi-square test was applied to intergroup comparisons of sex. Values of *p* < 0.05 were considered statistically significant.

Before the one or two-sample *t*-test, Jarque-Bera goodness-of-fit test was conducted to confirm the normal distribution of ALFF, ReHo, and FC maps (all voxels with *p* > 0.05). Subsequently, neuroimaging indices were analyzed using DPABI software. First, a one-sample *t*-test was performed to examine the functional activity and connectivity patterns in each group. Then, the *t*-statistics of group effect was analyzed after adjusting for covariates (including age, sex, and gray matter intensity and head motion parameter) with multiple linear regression. The test results were corrected for multiple comparisons with Gaussian random-field theory (GRF, voxel level *p* < 0.001, cluster level *p* < 0.05).

ALFF, ReHo values, and FC *z* scores in significantly altered brain regions were extracted for *Spearman* correlation analysis with pain intensity and neuropsychological data in BMP patients. We also explored the relationship of pain intensity with neuroimaging indices of each voxel (GRF corrected, voxel level *p* < 0.001, cluster level *p* < 0.05). The extracted values of both groups were used for receiver operating characteristic (ROC) curve analysis to evaluate their performance in discriminating individual subjects between the two groups. Leave-one-out cross-validation was performed to prevent possible inflated estimates of discriminant power and the validated AUC was obtained. Collinearity among neuroimaging indices was checked by using linear regression to avoid the loss in statistical power. And the probabilities of combined neuroimaging indices were obtained by using logistic regression in SPSS software and were used for the ROC curve analyses. The Delong test was applied to compare areas under ROC curves (AUCs) (20).

## Results

### Demographic and neuropsychological data comparisons

The BMP patients did not differ from the HCs in terms of age, sex, SAS, and SDS score (*p* > 0.05). The duration of pain in patients ranged from 20 to 2,095 days, and the pain score ranged from 1 to 7. Details are shown in Table 1.

### Functional activity and connectivity analysis

According to the one-sample *t*-test, both the BMP patients and HCs showed higher ALFF and ReHo than the global mean value mainly in the parietal and occipital lobes and lower ALFF and ReHo mainly in the frontal and temporal lobes. According to the two-sample *t*-test, BMP patients showed lower ALFF and ReHo mainly in the prefrontal cortex and higher ReHo in the bilateral thalamus and left fusiform gyrus. Higher ALFF values were not observed in BMP patients.

**Figure 1:**
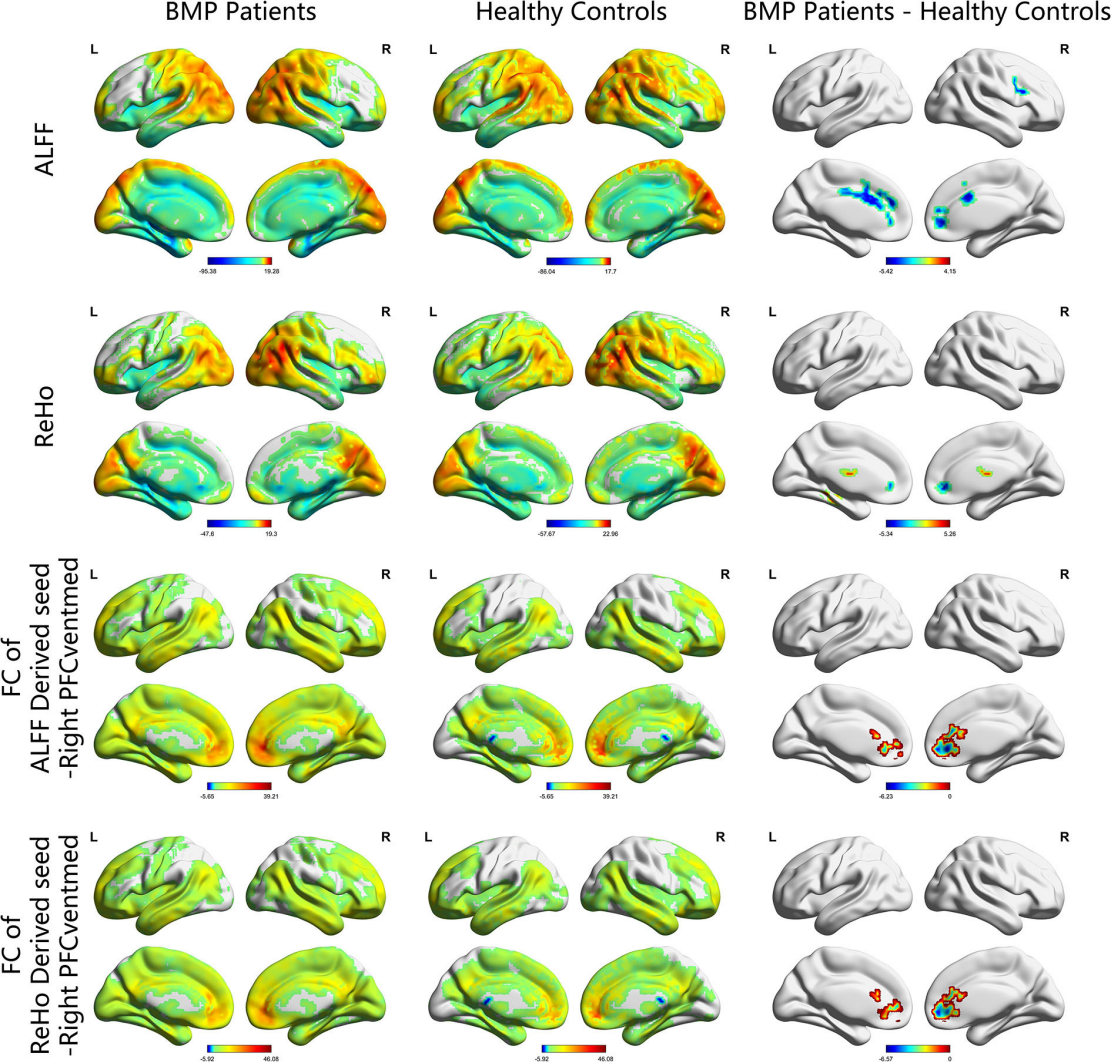
ALFF, ReHo, and FC maps of intragroup and intergroup comparisons. Corrected for multiple comparisons with Gaussian random-field theory (voxel level *p* < 0.001, cluster level *p* < 0.05). The color scale denotes the *t* value. ALFF, amplitude of low-frequency fluctuations; ReHo, regional homogeneity; FC, functional connectivity; PFCventmed, superior frontal gyrus, medial orbital; R, right; L, left.

**Figure 2:**
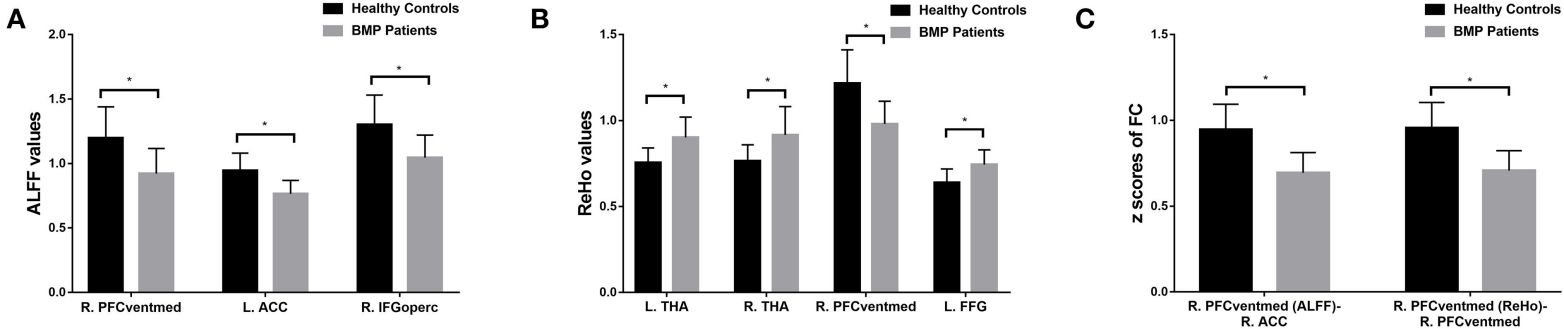
Brain regions with significant differences in ALFF **(A)**, ReHo **(B)**, and FC **(C)** between BMP patients and healthy controls. * *p* < 0.05 (corrected with Gaussian random-field theory, voxel level *p* < 0.001, cluster level *p* < 0.05). ALFF, amplitude of low-frequency fluctuations; ReHo, regional homogeneity; FC, functional connectivity; ACC, anterior cingulate cortex; IFGoperc, inferior frontal gyrus, opercular part; PFCventmed, superior frontal gyrus, medial orbital; THA, thalamus; FFG, fusiform gyrus; R, right; L, left.

According to the two-sample *t*-test, the brain regions with abnormal brain activity were set as the seed regions for FC calculation. In both groups, the right medial orbital of the superior frontal gyrus had higher FC than the medial frontal cortex and temporal lobes. Compared to HCs, BMP patients had lower FC of the right medial orbital of the superior frontal gyrus within the prefrontal cortex. The FC anchoring other seed regions showed no significant differences between the two groups. Details are shown in Table 2 and Figures 1, 2.

**Table 2:** Brain regions with aberrant ALFF, ReHo, and FC in BMP patients.

**Measurements**	**Brain regions**	**Hemisphere**	**BA**	**Peak MNI coordinates**			**Voxels**	***t*-Values**
				***x***	***y***	***z***		
ALFF	ACC	Left	24/32	0	12	30	294	−5.4158
	IFGoperc	Right	44	48	12	27	50	−4.5053
	PFCventmed	Right	10	6	48	−3	91	−4.8296
ReHo	THA	Left/Right	NA	−12	−18	15	98	5.2606
	FFG	Left	20	−36	−27	−18	54	5.0836
	PFCventmed	Right	10	6	45	−6	77	−5.3365
FC	Right PFCventmed (ALFF)-Right ACC	NA	11	6	39	−3	246	−6.2276
	Right PFCventmed (ReHo)-Right PFCventmed	NA	11	3	42	−3	262	−6.5679

ALFF, amplitude of low-frequency fluctuation; ReHo, regional homogeneity; FC, functional connectivity; ACC, anterior cingulate cortex; IFGoperc, inferior frontal gyrus, opercular part; PFCventmed, superior frontal gyrus, medial orbital; THA, thalamus; FFG, fusiform gyrus; BA, Brodmann area; NA, not applicable.

### Correlation and ROC curve analysis

**Figure 3:**
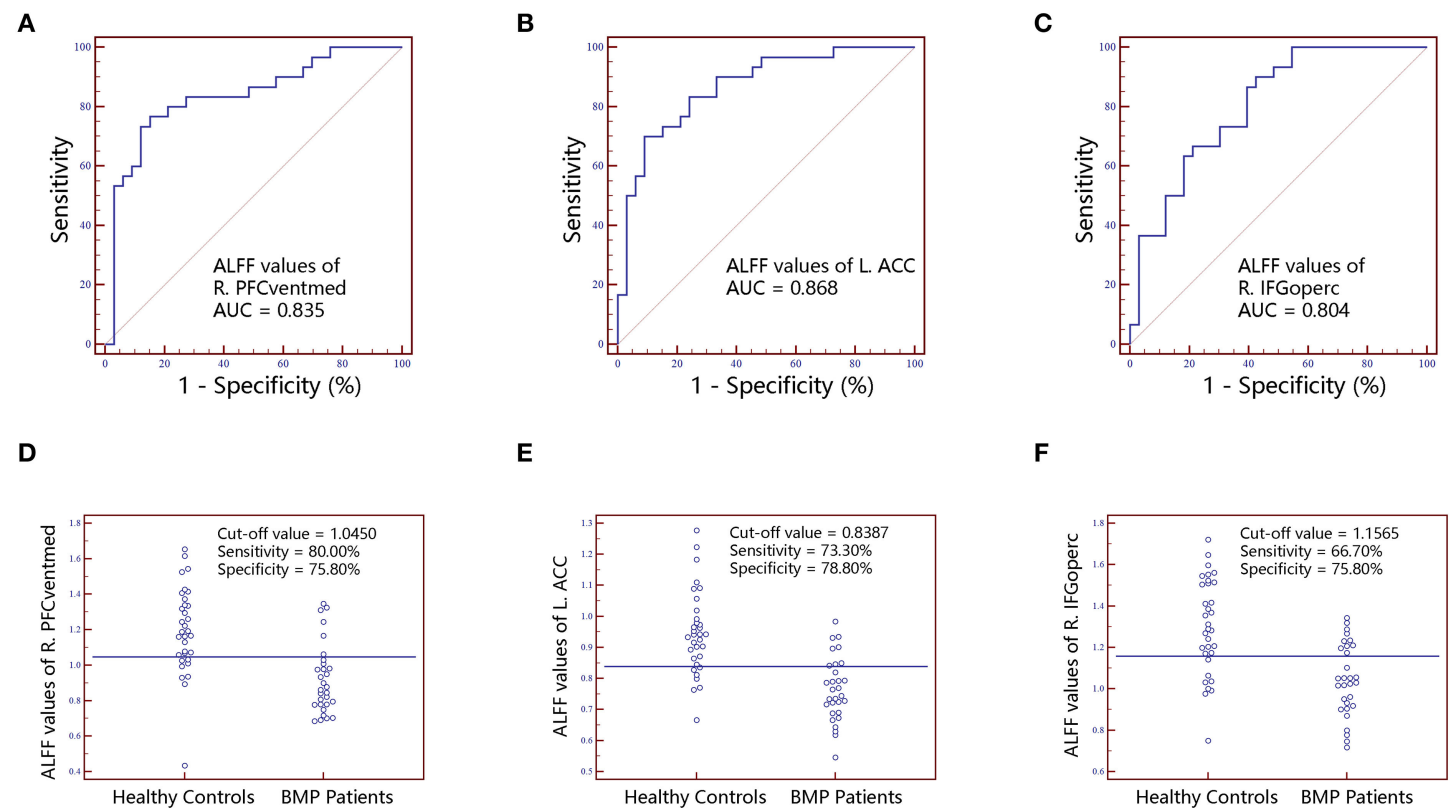
The classification performance of ALFF values extracted from individual brain regions identified by intergroup comparison. The top row are receiver operating characteristic curves for R.PFCventmed **(A)**, L.ACC **(B)** and R.IFGoperc **(C)**. The bottom row are interactive dot diagrams for R.PFCventmed **(D)**, L.ACC **(E)** and R.IFGoperc **(F)**. ALFF, amplitude of low-frequency fluctuations; ACC, anterior cingulate cortex; AUC, areas under receiver operating characteristic curve; IFGoperc, inferior frontal gyrus, opercular part; PFCventmed, superior frontal gyrus, medial orbital; R, right; L, left.

**Figure 4:**
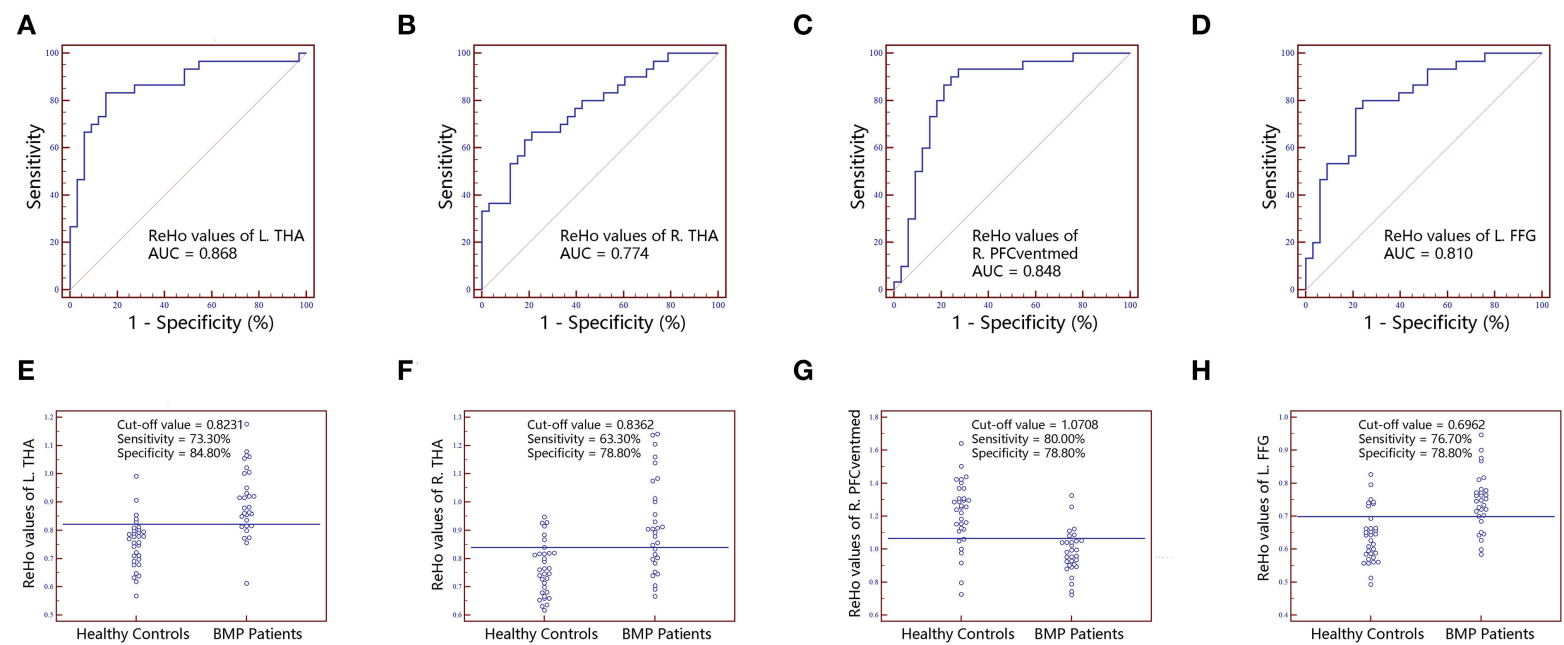
The classification performance of ReHo values extracted from individual brain regions identified by intergroup comparison. The top row are receiver operating characteristic curves for L.THA **(A)**, R.THA **(B)**, R.PFCventmed **(C)** and L.FFG **(D)**. The bottom row are interactive dot diagrams for L.THA **(E)**, R.THA **(F)**, R.PFCventmed **(G)** and L.FFG **(H)**. ReHo, regional homogeneity; AUC, areas under receiver operating characteristic curve; THA, thalamus; PFCventmed, superior frontal gyrus, medial orbital; FFG, fusiform gyrus; R, right; L, left.

**Figure 5:**
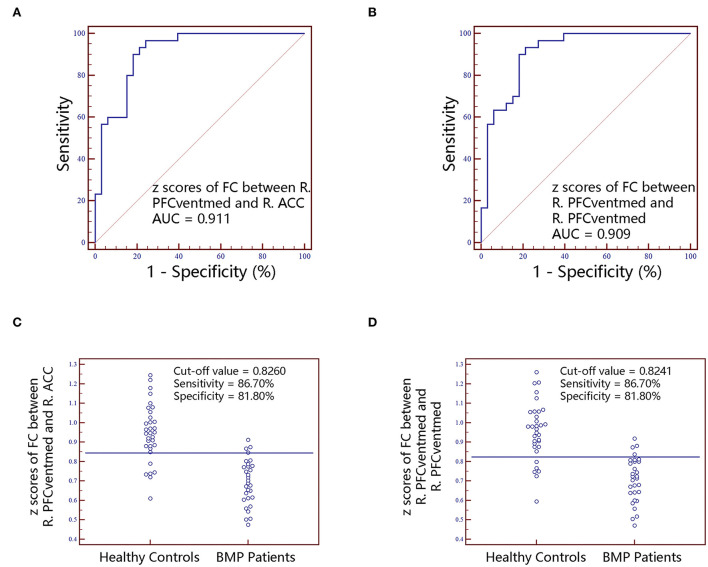
The classification performance of FC *z* scores extracted from individual brain regions identified by intergroup comparison. The top row are receiver operating characteristic curves for *z* scores of FC between R.PFCventmed and R.ACC **(A)** and *z* scores of FC between R.PFCventmed and R.PFCventmed **(B)**. The bottom row are interactive dot diagrams for *z* scores of FC between R.PFCventmed and R.ACC **(C)** and *z* scores of FC between R.PFCventmed and R.PFCventmed **(D)**. FC, functional connectivity; AUC, areas under receiver operating characteristic curve; ACC, anterior cingulate cortex; PFCventmed, superior frontal gyrus, medial orbital; R, right; L, left.

**Figure 6:**
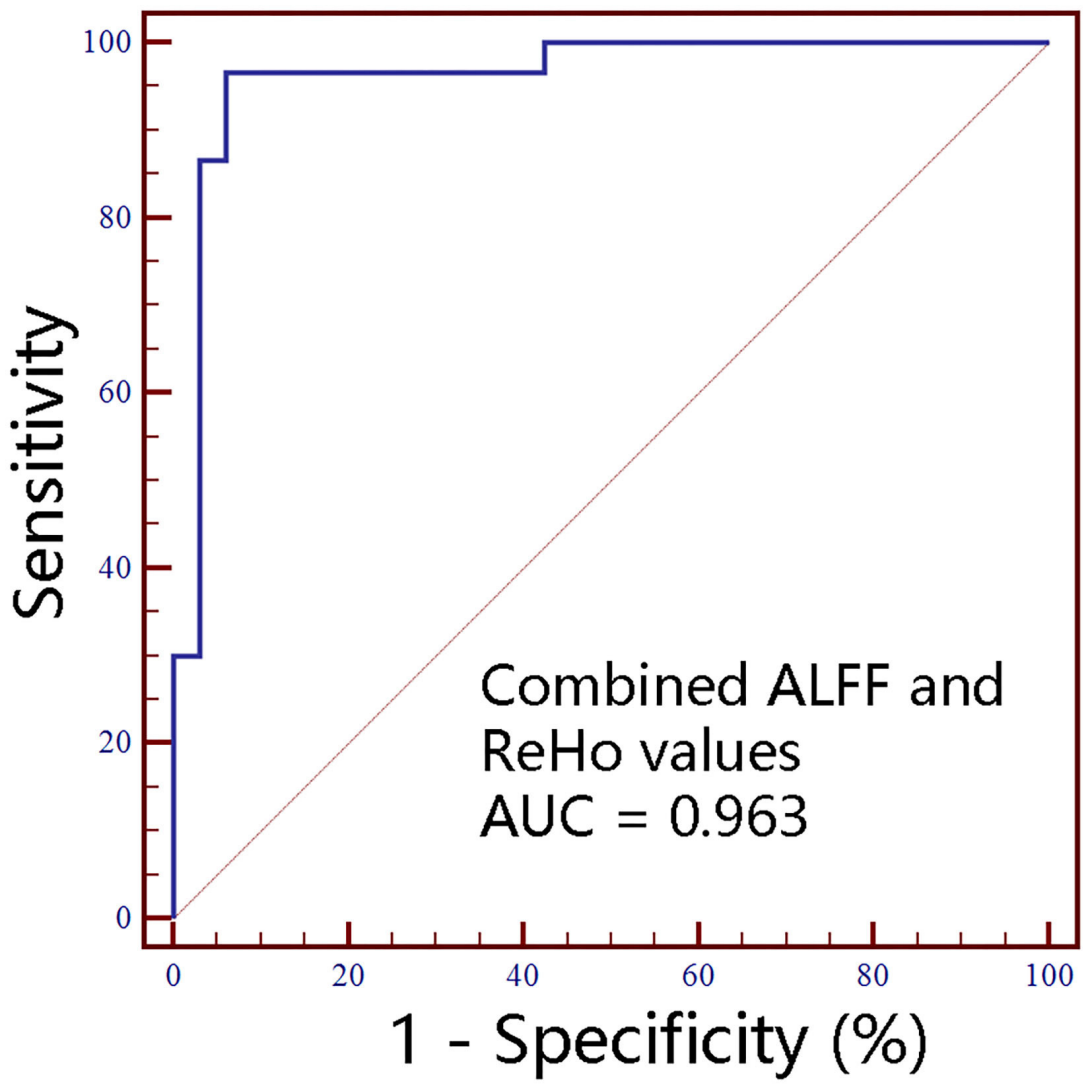
The classification performance of combined ALFF and ReHo values extracted from individual brain regions identified by intergroup comparison. ALFF, amplitude of low-frequency fluctuations; ReHo, regional homogeneity; AUC, areas under receiver operating characteristic curve; R, right; L, left.

No significant correlations between neuroimaging indices and pain scores or neuropsychological data were noted in BMP patients (*p* > 0.05; Table 3). The extracted values of ALFF from individual brain regions showed moderate discrimination performance as well as the values of ReHo, whereas the values of FC *z* scores showed better discrimination performance than ALFF or ReHo alone. The combined ALFF and ReHo values of all brain regions were superior to individual values in ROC analysis (*p* < 0.05). FC *z* scores were not involved in the combination due to their collinearity. The combined AUC of ALFF and ReHo showed no significant difference from the AUC of FC values (*p* > 0.05; Table 4, Figures 3–6).

**Table 3:** Results of *Spearman* correlation between neuroimaging indices and pain scores in BMP patients.

**Measurements**	**Brain regions**	**Hemisphere**	**ρ**	***p***
ALFF	ACC	Left	0.112	0.557
	IFGoperc	Right	0.140	0.459
	PFCventmed	Right	0.144	0.459
ReHo	THA	left	0.025	0.897
	THA	Right	−0.031	0.871
	FFG	left	−0.143	0.450
	PFCventmed	Right	−0.300	0.107
FC	Right PFCventmed (ALFF)-Right ACC	NA	−0.175	0.355
	Right PFCventmed (ReHo)-Right PFCventmed	NA	−0.186	0.326

BMP, bone metastasis pain; ALFF, amplitude of low-frequency fluctuations; ReHo, regional homogeneity; FC, functional connectivity; ACC, anterior cingulate; IFGoperc, inferior frontal gyrus, opercular part; PFCventmed, superior frontal gyrus, medial orbital; THA, thalamus; FFG, fusiform gyrus; NA, not applicable.

**Table 4:** Classification performance of neuroimaging indices in discriminating between BMP patients and healthy controls.

**Measurements**	**Brain regions**	**Hemisphere**	**AUC**	**Leave-one-out cross validated AUC**	**Cutoff values**	**Sensitivity (%)**	**Specificity (%)**	**Accuracy (%)**
ALFF	ACC	Left	0.868^a^	0.866	0.8387	73.30	78.80	76.20
	IFGoperc	Right	0.804^a^	0.802	1.1565	66.70	75.80	71.40
	PFCventmed	Right	0.835^a^	0.834	1.0450	80.00	75.80	77.80
ReHo	THA	Left	0.868^a^	0.867	0.8231	73.30	84.80	79.40
	THA	Right	0.774^a^	0.773	0.8362	63.30	78.80	71.40
	FFG	Left	0.810^a^	0.811	0.6962	76.70	78.80	77.80
	PFCventmed	Right	0.848^a^	0.848	1.0708	80.00	78.80	79.40
FC	Right PFCventmed (ALFF)-Right ACC	NA	0.911^b^	0.908	0.8260	86.70	81.80	84.10
	Right PFCventmed (ReHo)-Right PFCventmed	NA	0.909^b^	0.906	0.8241	86.70	81.80	84.10
Combined ALFF and ReHo values	Brain regions with significant intergroup difference of ALFF and ReHo	NA	0.963	0.961	NA	93.30	93.90	93.70

a Compared with combined ALFF and ReHo values, *p* < 0.05.

b Compared with combined ALFF and ReHo values, *p* > 0.05.

BMP, bone metastasis pain; ALFF, amplitude of low-frequency fluctuations; ReHo, regional homogeneity; FC, functional connectivity; ACC, anterior cingulate cortex; IFGoperc, inferior frontal gyrus, opercular part; PFCventmed, superior frontal gyrus, medial orbital; THA, thalamus; FFG, fusiform gyrus; NA, not applicable.

## Discussion

We investigated the brain functional activity and connectivity differences between BMP patients and HCs. Decreased ALFF and ReHo were found mainly in the frontal cortex, and increased ReHo was found in the thalamus and fusiform gyrus in BMP patients compared with HCs. Decreased FC within the prefrontal cortex was observed in BMP patients. These neuroimaging indices showed satisfactory discriminatory performance between the two groups and may serve as neuroimaging biomarkers.

The thalamus and prefrontal cortex (including the anterior cingulate cortex) have been repeatedly reported to be associated with chronic pain (21). As the relay station in ascending nociceptive inputs and descending pain modulatory pathways, the thalamus was reported to have functional and structural abnormalities in patients with migraines (22) and chronic low back pain (6). Blood oxygen level-dependent signal variability in the ascending trigeminal spinal-thalamo-cortical pathway was associated with headache severity (23). Therefore, the elevated activity of the thalamus in BMP patients may be driven by the transmission of pain information. Furthermore, the prefrontal cortex drives the cognitive modulation of the pain experience (15). Decreased pain-evoked brain activity in the prefrontal cortex was associated with reduced antinociceptive brain responses to pain in fibromyalgia patients (24). Increased prefrontal cortex activity was associated with reduced pain intensity after psychologic-based therapies in fibromyalgia patients (25). Therefore, the decreased brain activity and connectivity in the prefrontal cortex may suggest that BMP patients suffer from weakened antinociceptive cognitive modulation, which is consistent with previous studies on other types of pain.

A few studies have reported the possible role of the fusiform gyrus in pain processing. The fusiform gyrus anatomically connects the ventral visual network and is associated with facial recognition or face selection (26). Heightened activity in the fusiform gyrus was found to be a robust biomarker of pain intensity in experimental pain and chronic low back pain (27). The fusiform gyrus may be involved in the affective component of pain processing occurring in the amygdala and hippocampus by outputting the visualization of painful experiences (28). The present study's findings of increased activity in fusiform are consistent with previous studies and suggest the potential involvement of affective processing in BMP patients.

Neuroimaging indices have been used as biomarkers to discriminate between pain patients and controls. ALFF maps were used to classify chronic low back pain patients and controls with a moderate discriminate performance (accuracy rate >70%) (29). In addition, ReHo maps showed poor performance in classifying the more resilient chronic pain patients from the less resilient chronic pain patients (accuracy rate = 55%); however, the accuracy rate of combined ReHo and fractional ALFF achieved 79% (30). In the present study, the combined ALFF and ReHo showed satisfactory classification performance (accuracy rate = 93.7%) between BMP patients and HCs, which is comparable with the classification performance of FC maps. And the classification performance of FC maps was not significantly different from the combined ALFF and ReHo maps. Hence, ALFF, ReHo, and FC maps may serve as neuroimaging biomarkers for classifying BMP patients and HCs. However, the discriminant power of ROC may be overestimated by entering the values extracted from previously-identified significant brain regions, which can be inferred from the similarity between the original AUCs and leave-one-out cross-validated AUCs. Therefore, the classification performance in the present study should be interpreted with caution. In addition, the disadvantages of these neuroimaging indices are not negligible, including respiratory carbon dioxide influence on ALFF (31), the impact of neighborhood voxel size on ReHo (32), and the potential transitivity problem, controversial anti-correlations, and unsatisfactory reliability of FC (29, 33).

Of note, no correlation between brain activity or connectivity was found with pain intensity in the present study. The dissociation between the magnitude of the response in the brain and the persistence of pain may be due to the habituation of repetitive noxious stimuli (12). For instance, brain activity was significantly related to stimulus intensity rather than pain intensity in healthy participants during experimental tonic noxious heat stimulation (34). Moreover, a recent study suggests that fMRI may not be a reliable measure of reported pain intensity (35). Other confounding factors should also be taken into consideration, including pain caused directly by the tumor and treatment. On one hand, the tumor can release pain-modulating agents and their growth can erode into normal tissue, resulting in pain. On the other hand, surgical insult contributes to chronic post-surgical pain and chemotherapy and monoclonal antibodies can induce neuropathy which is associated with pain (36). In addition, the individual amplitude of pain may be facilitated by negative emotions (37), even though no significant differences in SAS and SDS scores were noted between BMP patients and HCs.

Some limitations should be acknowledged. First, the sample size is relatively small. The findings should be further validated in other datasets with larger sample sizes. Second, patients suffering from severe intensities of pain are impractical to be involved with potential evident head movement and only those with mild and moderate intensities were included. Therefore, bias is not avoidable. Third, we cannot exclude the confounding effects of analgesics, lung cancer, and its treatment. Fourth, the MRI data processing should be taken into consideration since there is no definite standard procedure. For instance, though the effects of variables of interest have remained largely after standardization (19), we must acknowledge that the choice of whether to conduct standardization may have an impact on the results. Despite these limitations, the preliminary positive findings warrant further investigation.

In conclusion, it is necessary to better understand cancer pain. Our findings suggested a decrease in brain functional activity mainly in the frontal cortex and an increase in the thalamus and fusiform gyrus in BMP. Moreover, a decrease in functional connectivity within the frontal cortex was observed. These abnormalities may be associated with the neuropathology of BMP and hold the potential to classify BMP patients and HCs. However, we should note that the present preliminary findings should be verified in future elaborate study designs.

## Data availability statement

The raw data supporting the conclusions of this article will be made available by the authors, without undue reservation.

## Ethics statement

The studies involving human participants were reviewed and approved by Medical Research Ethics Committee of Chongqing University Cancer Hospital. The patients/participants provided their written informed consent to participate in this study.

## Author contributions

DL contributed to the experiments, statistical analysis, and the writing of the manuscript. XZ contributed to the MRI data analysis. YT, HY, YC, LT, LY, SW, SL, JC, JL, and CW contributed to MRI and clinical data collection. HYQ and JZ are the guarantors of this study and had complete access to all the data in the study. All authors contributed to the article and approved the submitted version.

## Funding

The study was supported by the National Natural Science Foundation of China (82071883), Chongqing Medical Research Project of Combination of Science and Medicine (2021MSXM035), Graduate Research and Innovation Foundation of Chongqing, China (CYS21071), 2020 SKY Imaging Research Fund of the Chinese International Medical Foundation (Z-2014-07-2003-24), and Chongqing Natural Science Foundation (cstc2021jcyj-msxmX0319 and cstc2021jcyj-msxmX0313).

## Conflict of interest

The authors declare that the research was conducted in the absence of any commercial or financial relationships that could be construed as a potential conflict of interest.

## Publisher's note

All claims expressed in this article are solely those of the authors and do not necessarily represent those of their affiliated organizations, or those of the publisher, the editors and the reviewers. Any product that may be evaluated in this article, or claim that may be made by its manufacturer, is not guaranteed or endorsed by the publisher.
